# Disentangling true shape differences and experimenter bias: are dextral and sinistral snail shells exact mirror images?

**DOI:** 10.1111/j.1469-7998.2010.00729.x

**Published:** 2010-11

**Authors:** M Schilthuizen, M Haase

**Affiliations:** 1 National Museum of Natural History ‘Naturalis’Leiden, The Netherlands; 2 Institute for Tropical Biology and Conservation, Universiti Malaysia SabahKota Kinabalu, Malaysia; 3 Vogelwarte, Zoologisches Institut, Ernst-Moritz-Arndt-UniversitätGreifswald, Germany

**Keywords:** chirality, asymmetry, Pulmonata, Gastropoda, Mollusca, morphometrics, handedness

## Abstract

In theory, snails can come in two enantiomorphs: either dextral (coiling clockwise) or sinistral (coiling counter-clockwise). In snail species where both forms are actually present, coiling direction is determined by a single gene with delayed maternal inheritance; there is no predictable relationship between a snail's own coiling genotype and its actual coiling direction. Because of this genetic decoupling, it might be expected that dextral and sinistral individuals would be exact mirror images of one another. However, indications exist that there is a subtle but detectable shape difference between dextral and sinistral individuals that derive from the same gene pool. In this paper, we attempt to detect such differences in 50 dextral and 50 sinistral individuals of *Amphidromus inversus*, a species of land snail that is consistently chirally dimorphic. Four out of 18 volunteers who measured the shells with Vernier calipers found that sinistrals are stouter to a significant degree. A similar result was found by one out of five volunteers who measured the shells from photographs. These results do not allow distinguishing between real shape differences and a handling bias of sinistral as compared with dextral shells. However, when the same set of shells was subjected to a geometric morphometric analysis, we were able to show that sinistrals indeed exhibit a slight but significant widening and twisting of the shell near the palatal and parietal apertural areas. This result is surprising because species of the subgenus *Amphidromus* s. str. share a long history of chiral dimorphism, and the species would be expected to have been purged from disadvantageous interactions between direction of coil and general shell shape. We conclude that selection on the shape differences is either very weak or constrained by the fact that the pleiotropic effects of the chirality gene are of importance very early in development only.

## Introduction

Chirality (*handedness*) is the phenomenon in which a three-dimensional, asymmetric form can come in two mirror-image forms (McManus, 2002). Helical structures, in molecules (e.g. DNA and the α-helix of proteins) as well as in the bodies of living organisms (e.g. spirochaetes, the tendrils of vines and snail shells), display a well-known example of chirality (Asami, 1993). A helix may be coiled clockwise (dextral, right-handed) or anticlockwise (sinistral, left-handed); the two forms are mirror images of one another and cannot be superimposed. In snail shells, the difference in chirality of the spiral is easily seen by observing that in a dextral shell, when the apex is held up, the aperture is on the right-hand side of the shell, whereas in a sinistral shell, it is on the left (Gittenberger, 1988).

Coiling direction in snails (or, at least in pulmonate snails) is known to be determined by a single mendelian locus, with either the ‘dextral’ allele or the ‘sinistral’ allele being dominant (Schilthuizen & Davison, 2005; but see Utsuno & Asami (2010) for the discovery of a chirality randomizing gene in *Bradybaena*). The inheritance of the trait is, however, complicated by the fact that the gene is expressed not in the bearer itself, but in its offspring, if the bearer acts as the mother (so-called maternal inheritance; Boycott & Diver, 1923; Sturtevant, 1923; Diver, Boycott & Garstang, 1925;). This means that the expression of the gene is delayed by one generation compared with ‘normal’ genes. Although rare sinistral mutants are known for many dextral snail species and vice versa, the majority of snail species are directionally asymmetric, that is they are fixed for one coiling morph (usually the dextral one). In snails with internal fertilization, this directionality can generally be attributed to the inability of snails of opposite chirality to mate successfully, because the internal anatomy and even the courtship behaviour are reversed. This then leads to positive frequency-dependent selection against the rare morph, which buffers species against the establishment of that morph (Gittenberger, 1988; Asami, Cowie & Ohbayashi, 1998; Schilthuizen & Davison, 2005; Davison *et al.*, 2008;).

However, in snails in general, fitness loss may also be expected due to incompatibilities between the reversed chirality and the rest of the genomic and developmental environment, which has been selected for compatibility with the normal, non-reversed situation. Such pleiotropic effects are presumably the cause for the shell irregularities that have been reported in rare sinistral individuals of, for example *Cerion* (Gould, Young & Kasson, 1985), and probably relate partly to differences in early ontogeny between dextral and sinistral phenotypes, viz. a delayed onset of helical spindle inclination and spiral blastomere deformation in sinistrals (Shibazaki, Shimizu & Kuroda, 2004). In other snails, too, pleiotropic effects of chirality on the shell shape have been observed. In *Partula suturalis*, sinistral shells are shorter and squatter than dextral shells (Gould *et al.*, 1985; Johnson, 1987; Johnson, Murray & Clarke, 1993;). Asami (2001) reported on similar situations in *Achatinella*. Hendricks (2009) showed that the extinct sinistral *Conus adversarius* was morphologically more variable than the dextral congeneric species. In *Lymnaea stagnalis*, sinistral individuals showed, when compared with dextral ones with the same parental genomes, lower hatch rates, developmental aberrations and a more strongly expanded last whorl (Asami, 2007), and similar results were obtained by Utsuno & Asami (2007) for *Bradybaena*. Thus, in general, it appears that sinistral individuals from normally dextral snail species have relatively broader shells, although the differences can be very small, like in *P. suturalis*, for example, where the width/height ratio in sinistrals is just 1.5% (Davison *et al.*, 2009) to 2.6% (Johnson, 1987) greater. Making use of intrauterine offspring in preserved specimens of *P. suturalis* to determine the genotype, Davison *et al.* (2009) were able to compare the effects on shell shape of both a snail's coiling phenotype and its own coiling genotype, and found that the shell width/height ratio is determined by its coiling phenotype (hence by its mother's genotype), whereas its height is also determined by its own genotype. This means that if shell shape is under selection in a population, this will affect the population genetics of the chirality alleles via both maternal and classical Mendelian means (Kirkpatrick & Lande, 1989; Lande & Kirkpatrick, 1990;).

The south-east-Asian tree snail species *Amphidromus inversus* (and some 30 related species from the subgenus *Amphidromus*; Schilthuizen *et al.*, 2005; Craze, Elahan & Schilthuizen, 2006; Sutcharit & Panha, 2006;) is unusual among snails in that it displays balanced intra-population coil dimorphism close to equal proportions for dextrals and sinistrals. Field and molecular studies have shown that the dimorphism may be maintained by sexual selection actually *favouring* mates of the opposite chirality (Schilthuizen *et al.*, 2007; Schilthuizen & Looijestijn, 2009;), and that it is a phylogenetically old trait within the genus (Sutcharit, Asami & Panha, 2006). This would suggest that in *Amphidromus*, the long history of selection for chiral dimorphism should have purged populations from any ancestral disadvantageous pleiotropic effects of sinistrality (unlike *P. suturalis*, which shows chiral dimorphism only in a narrow zone flanked by large areas of fixed chirality where no such purging of deleterious effects would occur). Consequently, we would expect that dextral and sinistral *Amphidromus* shells are exact mirror images of one another and do not show the shape differences that appear to be present in species that are not normally dimorphic.

To test this prediction, we took shell measurements from a sample of sinistral and dextral individuals from a population of *A. inversus*. We initially took a single series of height and width measurements using Vernier calipers, a classical method for conchometry (Rensch, 1932; Peake, 1973; Teshima *et al.*, 2003; Davison *et al.*, 2009; see also Schilthuizen *et al.* (2007) for some preliminary biometrics on this particular set of *A. inversus* specimens). However, we quickly realized that this manual method introduces unexpected experimenter bias, where the handedness of the experimenter and his or her way of handling the calipers appear to influence the outcome to an unexpected degree. In this paper, we report on these biases, highlight the fact that they may have led to false positives in the conchometry literature and attempt to find methods in which they could be circumvented. In the end, we conclude that in *A. inversus*, there is, in fact, a true shape difference between dextrals and sinistrals, but that geometric morphometric methods are required to detect this unambiguously.

## Materials and methods

We collected 100 mostly fresh, adult shells of *A. inversus* (Gastropoda, Pulmonata, Camaenidae) from the localities ‘site 1’ and ‘site 2’ on the Malaysian island of Kapas (for details on these localities, see Schilthuizen *et al.*, 2007). At each locality, 25 sinistral and 25 dextral empty shells were collected. These four groups of 25 shells are hereafter termed S1, S2, D1 and D2. Each individual shell was given a unique number from 1 to 100.

**Figure 1:**
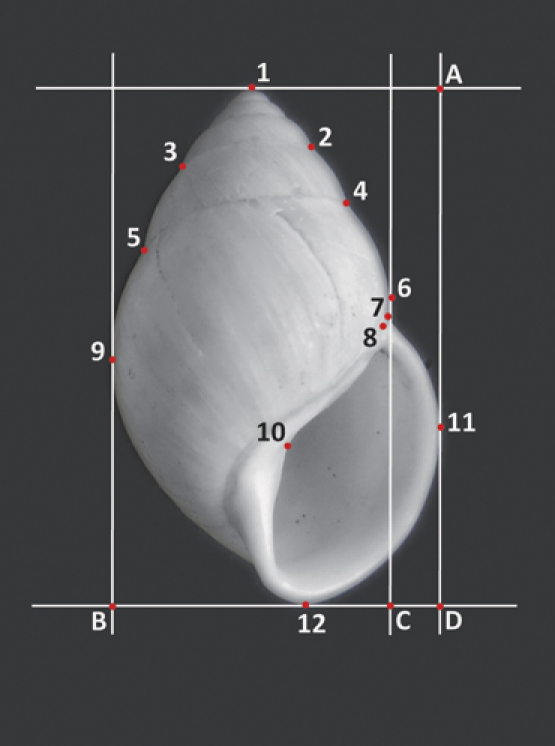
Conchometric landmarks and auxiliary lines. For the traditional conchometrics, SHEHEI=A–D; SHEWID=B–D; BODWID=B–C; ANGAPE=the angle between the lines 8–10 and 10–12. In the geometric morphometrics, landmarks 1–12 were used.

We then assembled a group of 18 volunteers, all biologists, but not all experienced malacologists, among whom five were left-handed and 13 were right-handed. Each volunteer was asked to take, to the nearest 0.1 mm, three conchometric measurements from each of the set of 100 shells [shell height (SHEHEI), shell width (SHEWID) and body whorl width (BODWID), as indicated in Fig. 1] using the same pair of Vernier calipers. The calipers were optimized for use by right-handed persons; however, this did not result in a greater variance in the measurements by the left-handers (data not shown). Most of the experimenters were observed and photographed while in the process of measuring. (Unfortunately, it was not possible to keep the volunteers unaware of the aim of the study, as was advocated by Gould, 1981.) We used a *t*-test for equality of means to test for univariate conchometric differences between dextral and sinistral individuals for the whole group of shells and we also applied a sequential Bonferroni correction (Rice, 1989) within each biometric.

We discovered curious inconsistencies among the experimenters and observed that dextral shells were handled differently from sinistral shells by at least some of the experimenters. To reduce this bias, we asked four volunteers to take digital photographs of the four sets of shells. The volunteers used a vertical photo stand, fixed to a worktop, with a Fuji Finepix S20pro camera (Fujifilm, Tokyo, Japan), and were asked to place the shells in such a way that the line of view was perpendicular to both the columella and the horizontal axis of the apertural plane. A scale bar was photographed along with each shell. The photographs of the sinistral shells were then mirrored in Adobe Photoshop, and the same four volunteers were asked to take the same three measurements once more, using Adobe Photoshop to measure the distances in pixels, and then using the scale bar to convert the measurements into mm.

Next, we attempted to reduce experimenter bias by having an experienced biometrician (M. H.) use the photographic method and testing repeatability. All the procedures that follow were carried out by M. H. within a period of 1 week.

Although performed in a different lab, the photographic method was essentially the same as before. Shells were balanced on a slightly concave socket of Styrofoam and positioned in such a way that the line of view was perpendicular to both the columella and the horizontal axis of the apertural plane. A Nikon D-70s camera (Nikon Corp., Tokyo, Japan) equipped with an AF Nikkor 28–105 mm fixed on a stand was used to photograph each specimen at the same scale. Photographs of sinistral shells were again mirrored in Adobe Photoshop. Images were transformed into a tps format using tpsUtil (Rohlf, 2004*a*) and landmarks were defined in tpsDig 2.0 (Rohlf, 2004*b*). To assess repeatability of the measurements (using *t*-tests), the procedure described above was carried out twice on the same set of shells, once on 30 April 2009 and again on 2 May 2009. Using auxiliary lines parallel and perpendicular to the columella, a set of landmarks was placed on the image, consisting of four points on the intersections of the auxiliary lines, and three more points on either side of the parietal and columellar edges of the aperture (Fig. 1). These points were used to calculate SHEHEI (distance A–D), SHEWID (distance B–D), BODWID (distance B–C), the angle between lines 8–10 and 10–12 (ANGAPE) as well as centroid size as a proxy for the overall size, using the program TMorphGen6c from the IMP suite of programs by Sheets and colleagues (http://www3.canisius.edu/~sheets/morphsoft.html). Repeatability between sessions was high (*P*>0.80 for all comparisons).

We used data from both sessions to perform analyses for SHEHEI, SHEWID, BODWID and ANGAPE: first, analyses of variance for the four groups D1, D2, S1 and S2, then *t*-tests to compare all sinistrals (from sites 1 and 2 combined) with all dextrals (from sites 1 and 2 combined), and finally, *t*-tests to compare all individuals from site 1 (dextrals and sinistrals combined) with those from site 2 (dextrals and sinistrals combined). ANOVAs and *t*-tests were carried out in past 1.81 (Hammer, Harper & Ryan, 2001).

Although repeatability of M. H.'s approach of measuring from photographs was high, experimenter bias could not be excluded. Using geometric morphometrics based on several landmarks placed directly on the shells, it should be possible to distinguish a systematic handling bias resulting in slight rotation around one or more axes from true shape differences. In the former case, displacements of landmarks should all have the same direction, and the closer a landmark to the periphery of the shell, the longer the vectors.

For the geometric morphometrics, the set of landmarks outlined above was augmented with additional ones as shown in Fig. 1. This resulted in the second set of landmarks: 1–12. Of these, numbers 1, 8 and 10 were of type 1, numbers 2–5 and 7 were of type 2 and the others were of type 3 (Bookstein, 1991). Analyses were performed again with programs from the IMP suite. Procrustes superimpositions were generated in CoordGen6h. We used TwoGroup6h to test the repeatability between both measurement sessions and found that they could not be distinguished (Goodall's *F*-tests, *P*>0.58 in all five comparisons), indicating good repeatability. With the same program, pair-wise comparisons of sites and coiling morphs were performed and graphical representations of differences between means as vectors of landmark displacement on thin-plate splines were generated. Canonical variates analyses (CVA) were carried out in CVAgen6n as a basis for assignment tests including all shells or shells from either site, respectively.

At one point earlier in the series of tests just described, one shell from group D1 was lost. For this reason, we decided to remove this shell from all previous datasets, so that all datasets would be comparable, and derived from the same set of 99 shells.

## Results

**Figure 2:**
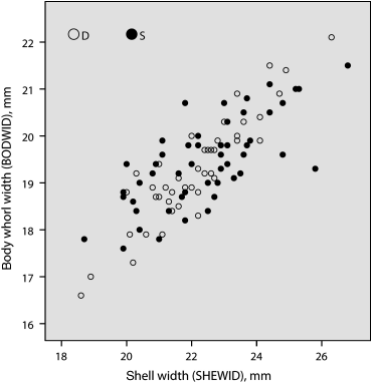
Scatter plot of caliper measurements for SHEWID and BODWID recorded by volunteer V2; these data do not reveal a significant difference between dextrals and sinistrals for either biometric.

**Figure 3:**
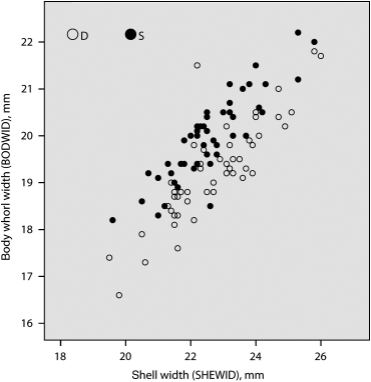
Scatter plot of caliper measurements for SHEWID and BODWID recorded by volunteer V3; these data reveal a significant difference (*P*=0.001; *t*-test) between dextrals and sinistrals for BODWID.

**Figure 4:**
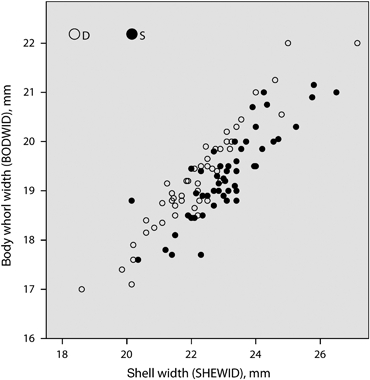
Scatter plot of caliper measurements for SHEWID and BODWID recorded by volunteer V10; these data reveal a significant difference (*P*=0.002; *t*-test) between dextrals and sinistrals for SHEWID.

Our 18 sets of caliper measurements (Table 1) showed curious inconsistencies. None of the volunteers recorded a significant difference in shell height between dextral and sinistral shells. For body whorl width, two volunteers found significantly (*P*=0.001) higher values for sinistral shells (mean difference=0.7 mm); all 16 other volunteers found no significant difference in body whorl width. For shell width, more than half (10 out of 18) of the volunteers found greater values for sinistral shells compared with dextral ones. Four of these differences (mean difference=1.0 mm) remained significant after sequential Bonferroni's correction. This group of four volunteers did not overlap with the two who found a body whorl width difference. As an illustration of these results, Figs 2–4 show scatter plots of very divergent BODWID and SHEWID results obtained by three selected volunteers. The overall trends, however, were that sinistral shells were measured to be stouter than dextral ones: 16 out of 18 volunteers found higher mean values in sinistrals for both BODWID and SHEWID.

**Table 1:** Means and standard deviations for each of the three conchometrics SHEHEI, SHEWID, and BODWID, as measured with vernier calipers by each of the 18 volunteers (shaded values indicate the larger of the comparison)

Volunteer	SHEHEI S (sd)	SHEHEI D (sd)	Significance	SHEWID S (sd)	SHEWID D (sd)	Sign.	BODWID S (sd)	BODWID D (sd)	Significance
V1	37.99 (2.43)	37.41 (3.62)	NS	22.95 (1.22)	22.30 (1.54)	*P*=0.020	19.37 (0.87)	19.06 (1.13)	NS
V2	38.29 (2.40)	37.47 (3.64)	NS	22.42 (1.75)	21.97 (1.56)	NS	19.46 (0.93)	19.22 (1.10)	NS
V3	38.37 (2.38)	37.47 (3.58)	NS	22.59 (1.23)	22.71 (1.44)	NS	19.94 (0.90)	19.26 (1.09)	*P*=0.001
V4	38.29 (2.47)	37.28 (3.56)	NS	23.59 (1.45)	23.64 (2.20)	NS	20.84 (1.17)	20.50 (1.62)	NS
V5	38.32 (2.43)	37.37 (3.73)	NS	23.14 (1.30)	22.11 (1.60)	*P*=0.001	19.98 (1.00)	19.68 (1.22)	NS
V6	38.19 (2.41)	37.46 (3.61)	NS	23.15 (1.20)	22.53 (1.32)	*P*=0.017	22.28 (1.86)	21.53 (1.83)	NS
V7	38.00 (2.48)	37.40 (3,60)	NS	22.90 (1.25)	22.50 (1.55)	NS	19.60 (0.93)	19.40 (1.11)	NS
V8	38.27 (2.43)	37.58 (3.66)	NS	23.21 (1.31)	22.55 (1.61)	*P*=0.027	19.55 (0.99)	19.52 (1.20)	NS
V9	38.02 (2.40)	37.19 (4.16)	NS	23.29 (1.21)	22.22 (1.49)	*P*=0.000	19.54 (0.89)	19.11 (1.12)	NS
V10	38.27 (2.44)	37.41 (3.66)	NS	23.13 (1.26)	22.24 (1.47)	*P*=0.002	19.33 (0.88)	19.28 (1.09)	NS
V11	38.20 (2.42)	37.40 (3.65)	NS	21.00 (1.12)	20.60 (1.40)	NS	19.20 (1.19)	19.00 (1.21)	NS
V12	38.33 (2.44)	37.54 (3.61)	NS	22.59 (1.33)	22.35 (1.58)	NS	18.98 (1.01)	19.25 (1.18)	NS
V13	38.30 (2.54)	37.40 (3.65)	NS	22.40 (1.44)	21.70 (1.60)	*P*=0.020	19.10 (1.00)	19.00 (1.21)	NS
V14	38.14 (2.45)	37.31 (3.66)	NS	23.24 (1.30)	22.65 (1.49)	*P*=0.039	19.73 (0.95)	19.04 (1.14)	*P*=0.001
V15	38.10 (2.37)	37.55 (3.63)	NS	23.10 (1.51)	22.00 (1.79)	*P*=0.001	19.69 (0.87)	19.50 (1.07)	NS
V16	38.31 (2.45)	37.37 (3.60)	NS	22.75 (1.35)	22.22 (2.44)	NS	19.14 (0.79)	19.16 (1.07)	NS
V17	38.10 (2.39)	37.30 (3,53)	NS	22.80 (1.20)	22.50 (1.47)	NS	19.70 (0.96)	19.50 (1.15)	NS
V18	34.90 (2.47)	34.54 (3.70)	NS	20.86 (1.27)	20.31 (1.47)	*P*=0.050	17.53 (1.00)	17.11 (1.26)	NS

Significance was determined with *t*-tests.

Observing the volunteers during their handling of shells and calipers revealed differences in the manner of measuring. For example, for SHEWID measurement, most volunteers held the shell aperture towards the calipers, but volunteers V06, V08, V11 and V16 held the shell apex towards them, while one of these (V06) was exceptional in doing this with the cervical area towards the experimenter; all others held the apertural area towards themselves during the measurement. Some volunteers measured dextral and sinistral shells in consistently different ways. For example when measuring SHEHEI, V12 held dextral shells with the cervical area towards the experimenter, but sinistral shells with the aperture towards the experimenter.

These idiosyncrasies were removed by letting four volunteers take measurements from digital photographs in which sinistral shells had been mirrored to make them appear dextral. The measurements taken by these volunteers (Table 2) revealed that some, but not all, experimenters again found significantly greater values for SHEWID and BODWID in the sinistral shells; only one value per biometric remained significant after sequential Bonferroni's correction. Curiously, in contrast with the caliper measurements, significant differences were found more prominently in BODWID than in SHEWID, and there was no apparent correspondence between a volunteer's caliper results and his/her photo results.

**Table 2:** Means and standard deviations for each of the three conchometrics SHEHEI, SHEWID, and BODWID, as measured from photographs by each of four volunteers (shaded values indicate the larger of the comparison)

Volunteer	Gender	Handedness	SHEHEI S (sd)	SHEHEI D (sd)	Sign.	SHEWID S (sd)	SHEWID D (sd)	Sign.	BODWID S (sd)	BODWID D (sd)	Sign.
V7	F	Right	39.12 (2.68)	37.98 (3.91)	NS	23.99 (1.45)	23.49 (1.90)	0.049	20.17 (1.03)	19.89 (1.85)	0.031
V11	M	Right	39.97 (2.67)	38.35 (3.89)	NS	24.32 (1.55)	23.65 (1.99)	0.020	20.34 (1.06)	19.93 (1.89)	0.008
V13	M	Right	40.17 (2.74)	38.95 (4.06)	NS	24.40 (1.54)	23.92 (1.96)	N.S.	20.73 (1.16)	20.52 (1.88)	N.S.
V17	M	Right	39.22 (2.68)	38.14 (3.96)	NS	24.01 (1.43)	23.57 (1.94)	N.S.	20.23 (0.99)	19.99 (1.86)	0.047

Significance was determined with *t*-tests.

The ANOVAs for both sessions of measurements taken by M. H. showed significant or near-significant differences between S1 (sinistrals from site 1) and D2 (dextral from site 2) in all biometrics except APEANG, which appeared to result from S1 being overall larger than D2 [centroid size was significantly greater (*P*=0.03 and 0.02, respectively) in both sessions]. *T*-tests to compare all sinistrals (from sites 1 and 2 combined) with all dextrals (from sites 1 and 2 combined) revealed no differences, whereas *t*-tests to compare all individuals from site 1 (dextral and sinistrals combined) with those from site 2 (dextral and sinistrals combined) again showed a size difference, with biometrics SHEHEI, SHEWID, BODWID and centroid size (but not ANGAPE) being significantly greater for individuals from site 1 compared with those from site 2.

**Figure 5:**
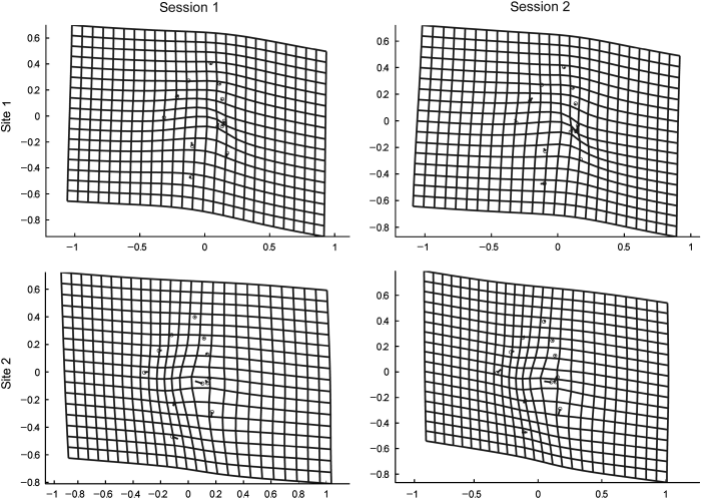
Deformation grids and vectors indicating landmark transformations from the mean dextral to the mean sinistral shell (exaggeration five times).

When the same set of landmarks was augmented with additional ones and subjected to geometric morphometrics, however, more subtle shape differences were revealed. Table 3 summarizes the results, which show that strongly significant shape differences existed between sites 1 and 2 as well as between dextrals and sinistrals, both within sites and across sites. The shape differences between dextrals and sinistrals and the high repeatability is illustrated in Fig. 5. At both sites, transforming one coiling morph into the other imposed the strongest distortions in the top corner of the aperture, however, with direction differing between sites. The CVA distinguishing sinistrals and dextrals from both sites assigned 60.6% of individual shells correctly. In within-site comparisons, this percentage improved considerably to 85.7% (site 1) and 86% (site 2), respectively.

**Table 3:** Pairwise shape comparisons in the framework of geometric morphometrics based on Goodall's *F*-tests. In each box, the top value is for session1, the lower value is for session 2

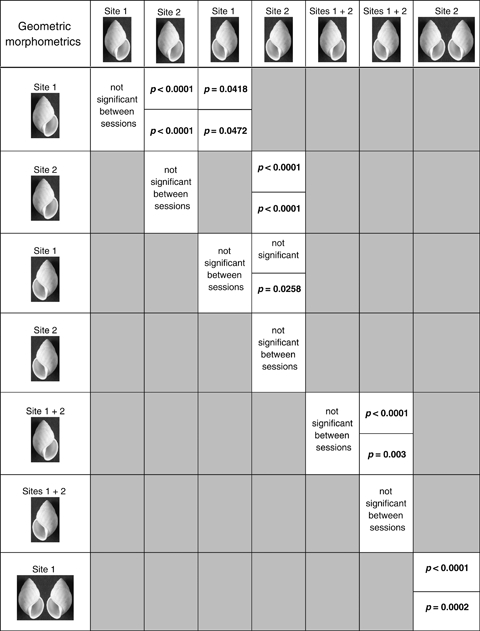

## Discussion

Vernier calipers have been used in malacology traditionally and extensively for measuring the shell shape manually and for detecting differences at various taxonomic levels (Goodfriend, 1986), that is between snail species (e.g. Cabral, 2003; Jordaens, Van Riel & Backeljau, 2003;), between snail populations (e.g. Solem, 1955; Seeley, 1986; Baminger & Haase, 2000; Van Riel *et al.*, 2001;) and within populations (e.g. Wolda, 1969; Heller & Farstay, 1989; Vinarski, 2007;). The limitations of the method are well known: measurement error is sometimes large relative to the true differences, which is why experimenters often remeasure multiple times and average across these (e.g. Solem, 1955); and individual variation between experimenters is routinely countered by separating datasets that were taken by different experimenters (e.g. Davison *et al.*, 2009). The present study, however, revealed that, in addition to random errors, systematic errors may be introduced into caliper-derived sets of data when experimenters handle the two enantiomers of helical objects (in this case: snail shells) differently, leading to conspicuously divergent results for different measurers. When attempting to detect subtle shape differences between dextral and sinistral individuals from the same species or even the same population, this bias can pose a serious problem. Many of the studies published on the influence of coiling direction on intraspecific snail shell shape variation so far might have been subject to this problem.

In *P. suturalis* and *P. otaheitana*, Crampton (1916, 1932) used caliper measurements to generate shell height and width data. These data were analysed by Gould *et al.* (1985) to reveal an overall tendency for sinistrals to be stouter than dextral. Johnson (1987) pointed out that Crampton's data were often combinations of multiple demes and proceeded to examine (presumably also with caliper measurements) 18 separate populations of *P. suturalis*, which confirmed the association of sinistrality with stouter shells. Davison *et al.* (2009) used caliper measurements, taken by a single right-handed experimenter from 1016 *P. suturalis* shells from 22 separate populations, and showed a significant trend for sinistrals to be stouter. This trend was confirmed by a second, left-handed experimenter, a measure that was prompted by unpublished versions of the present paper. In *Cerion*, Gould *et al.* (1985) used calipers to take measurements from five of the six known sinistral specimens of *C. incanum* (two shells) and *C. glans* (three shells) and found that the sinistrals were of a shape – involving relatively small apertures and a slight twist in the axis of coiling – that was normally only found in extreme dextral individuals.

In all the studies mentioned above, slight but significant shape differences were detected between sinistrals and dextrals. Mostly, as far as can be judged from the information provided in the respective papers, the caliper measurements were taken by a single individual. The confusing mix of results produced by our 18 measurers suggests that such results should be treated with caution. Some of our volunteers found highly significant differences that were of the same order of magnitude as those reported in the literature for other chirally dimorphic species, whereas other individuals, using the same shells and the same tools, found no difference whatsoever. This shows that a single set of caliper-derived data may reflect real shape differences as likely as measurer bias.

To avoid this potential problem, some studies (Gould *et al.*, 1985; Asami, 2007; Davison *et al.*, 2009;) have used additional measurements from photographs. The results obtained in this way by Davison *et al.* (2009) confirmed their caliper-based results. In *Lymnaea stagnalis* individuals with the same parental genomes, dextrals have spire whorls that are translated largely along the coiling axis, while sinistrals translate and expand the last whorl (Asami, 2007). These shape differences were reflected in linear metrics up to 10% different between *L. stagnalis* coiling morphs. Our results, obtained by having four volunteers place shells under a camera, digitally photographing them, mirroring images of sinistrals to make them appear dextral and then measuring distances in the images, again produced conflicting results. Some volunteers found significant differences, but the results were less strongly divergent than with the calipers measurements, and after sequential Bonferroni's correction, only the BODWID data obtained by one volunteer remained significant. This might suggest that the significant results obtained with the more error-prone caliper method were all due to experimenter bias and that there is no real shape difference. However, it should be pointed out that the photographic method is also not free of bias, as the placement of a shell under the camera requires handling it and, unbeknownst to the volunteer, he or she might position dextrals and sinistrals slightly differently. So, even with the photograph method, it might be impossible to separate real shape difference from systematic experimenter error.

In principle, the geometric morphometric method, which also used photographs taken by manually positioning shells under a camera, might seem to suffer from the same methodological dilemma. However, because the method allows the vectors representing shape differences to be visualized, it might be possible here to distinguish true from introduced shape differences, as follows. Our geometric morphometric results show that the significant shape difference between dextrals compared with sinistrals is due to a widening and twisting of the shell near the palatal and/or parietal sides of the aperture. If these differences were due to chirally biased, experimenter-induced rotation of the shell along its vertical axis during the positioning of the shell under the camera, the vectors around this area of the shell would be oriented similarly. The thin-plate splines, however, show that the vectors are directed very differently. This can only be explained by their representing, at least partly, true shape differences between dextrals and sinistrals. Although many more manipulative steps are necessary in generating landmark data in contrast to caliper measurements, repeatability proved to be very high (see Haase & Misof, 2009). The principal reason may be that positioning the shell, which probably introduces the highest variance, is done only once, whereas in the manual measuring approach, the shell is re-positioned for each measurement taken. This may also be a reason why taking measurements from photographs yielded more balanced results.

Taken together, our results, then, confirm the reports from other snail species that dextrals and sinistrals from the same genetic background are not exact mirror images of one another. In *A. inversus* from Kapas, this difference is reflected in a widening and twisting of the parietal and/or palatal apertural area in sinistrals (though, interestingly, in somewhat different ways in both sites), which probably explains the often greater values for BODWID and SHEWID recorded by our volunteers, as these conchometrics are anchored at the parietal and palatal sides of the aperture, respectively. In this respect, the shape difference is similar to the shape differences reported from other chirally dimorphic snails. However, in view of the difficulty in detecting these subtle differences with calipers or from two-dimensional projections, we suggest that future work in this area explore conchometry by three-dimensional scanning of shells.

That a shape difference should be present between dextral and sinistral *Amphidromus* is in itself somewhat surprising. By studying parent and offspring chirality genotypes in *P. suturalis*, Davison *et al.* (2009) proved that shell shape is affected by an interaction between a snail's coiling direction (maternally determined) and its own genotype. Because shell shape is usually finely tuned to the environment (Cook & Jaffar, 1984; Goodfriend, 1986;), it may be expected that selection will remove such interactions (assuming they are disadvantageous) in species such as those of *Amphidromus* s. str., which have a long history of intrapopulational chiral dimorphism (Sutcharit *et al.*, 2006). That this has not happened may indicate an extremely weak selection, the fact that selection is less effective because of the reduced (as maternally delayed) heritability (Schilthuizen *et al.*, 2007) or, perhaps more likely, a developmental constraint (sensu Gould & Lewontin, 1979), given that the chirality locus acts very early in development (Shibazaki *et al.*, 2004; Davison *et al.*, 2009;). We suggest that further studies of the maintenance of chiral dimorphism in *Amphidromus* take these effects into account.
